# Preparing ethical review systems for emergencies: next steps

**DOI:** 10.1186/s12910-023-00957-2

**Published:** 2023-10-27

**Authors:** Katharine Wright, Nic Aagaard, Amr Yusuf Ali, Caesar Atuire, Michael Campbell, Katherine Littler, Ahmed Mandil, Roli Mathur, Joseph Okeibunor, Andreas Reis, Maria Alexandra Ribeiro, Carla Saenz, Mamello Sekhoacha, Ehsan Shamsi Gooshki, Jerome Amir Singh, Ross Upshur

**Affiliations:** 1 WHO HQ, Geneva, Switzerland; 2 Ministry of Health, Wellington, New Zealand; 3 Ministry of Health and Population, Cairo, Egypt; 4 University of Ghana, Accra, Ghana; 5 University of Oxford, Oxford, UK; 6 Research Ethics Committee, The University of the West Indies – Cave Hill, Cave Hill, Barbados; 7 High Institute of Public Health, Alexandria, Egypt; 8 Formerly: WHO EMRO, Cairo, Egypt; 9 Indian Council of Medical Research, Bengaluru, India; 10 WHO AFRO, Brazzaville, Republic of Congo; 11 National Ethics Committee for Clinical Research (CEIC), Lisbon, Portugal; 12 WHO / Pan American Health Organization, Washington, DC USA; 13 National Health Research Ethics Council, Pretoria, South Africa; 14 Medical Ethics and History of Medicine Research Center, Tehran University of Medical Sciences, Tehran, Iran; 15 Monash Bioethics Center, Monash University, Melbourne, Australia; 16 University of KwaZulu-Natal, Durban, South Africa; 17 Dalla Lana School of Public Health, University of Toronto, Toronto, Canada

**Keywords:** Ethical preparedness; research ethics; ethical review; rapid review; COVID-19; infectious disease outbreaks; low- and middle-income countries

## Abstract

Ethical review systems need to build on their experiences of COVID-19 research to enhance their preparedness for future pandemics. Recommendations from representatives from over twenty countries include: improving relationships across the research ecosystem; demonstrating willingness to reform and adapt systems and processes; and making the case robustly for better resourcing.

## Ethical preparedness and ethical review

There is a growing recognition that being ‘ethically prepared’ is an important part of emergency preparedness, by helping facilitate an effective and ethical research-led response to pandemics [1, 2]. This includes preparedness of the ethical review system: at local, national, regional, and global level; and across multiple domains including the capacities of committee systems, staff and members. In recognition of the key role within research ecosystems played by ethical review systems during emergencies, in September 2022 the Health Ethics and Governance Unit of the World Health Organization (WHO) brought together representatives from over twenty countries at a pre-workshop to the Global Summit of National Ethics Committees in Lisbon, Portugal. Attendees took stock of their collective experiences of ethical review during COVID-19 and other epidemics, and explored how better to prepare for emergencies in the future. In this paper, we present the key findings and recommendations that resulted from that meeting. The discussion at the meeting was largely concerned with national-level policies and experiences without distinguishing between different forms of research. By implication, specific references to research generally related to clinical trials, although other kinds of research were briefly touched upon by some speakers, and the findings and recommendations should be read in that light. Further exploration is required to understand ethics committees’ experiences of diverse research approaches, and how these are integrated, both in emergency and non-emergency contexts.

## Learning from past emergencies

In the years preceding the emergence of COVID-19, substantial progress was made in planning for flexible and effective ethical review of research during emergencies, building on experiences in previous outbreaks including SARS, H1N1 pandemic influenza, Ebola and Zika. Detailed guidance and recommendations were produced targeting both global [1, 3, 4] and regional [5] audiences; in many cases these were developed in partnership with those facing these ethical challenges on the ground including members of national ethics committees, the ALERRT clinical research network, and ethicists participating in the Zika Ethics Consultation initiated by the Pan American Health Organization (PAHO). Training materials addressing emergency contexts were produced to support capacity strengthening within individual research ethics committees [6, 7], as were indicators to promote and support a more strategic approach to ethics systems at national level [8].

This work paid dividends in early 2020 as the nature and extent of the COVID-19 pandemic became clear. Leadership from WHO at both headquarters [9] and regional [10] level resulted in the rapid production of COVID-19-specific ethical guidance and materials, with the important role played by research ethics committees in emergency response also recognised by key regional bodies [11]. WHO’s own processes of ethical review at headquarters and regional level were also rapidly adapted to expedite the review of priority studies. Speakers at the meeting noted how Regional Offices played an important facilitative role behind the scenes, for example in helping tackle bottlenecks in systems, and promoting dialogue with other stakeholders in healthcare such as traditional medicine practitioners. In some countries, national research ethics bodies took a leading role in rapidly developing systems, guidance and support in response to their own countries’ needs.

## What went well as a result of initiatives to promote ethical preparedness

Attendees at the Lisbon meeting shared examples of how these elements of ethical preparedness had helped support good practice in their own countries as they sought to respond to the unprecedented challenges of the COVID-19 pandemic (see Table 1). In addition to the development of flexible, responsive review procedures at the level of individual committees, contributors emphasised the importance of rapidly establishing national strategies to clarify the respective roles and responsibilities of different levels of committee and their relationship with other parts of the research oversight system [12].

**Table 1:** Examples of how responsive and effective ethical review was achieved for COVID-19 studies, shared by attendees at the Lisbon meeting

At the national level**, the rapid development of national strategies** played a key role in facilitating effective ethical review, including through setting out the respective roles of different levels of committee, and providing procedural and substantive guidance for individual ethics committees. Brazil and Panama, for example, rapidly implemented national strategies for allocating review requests, and then adjusted these in the light of experience. Brazil retained all clinical trials for review at national level without prior local review; Panama initially reviewed all COVID-19 studies centrally but then shifted to a system of delegation for studies considered less high risk. New Zealand had systems up and running within two weeks, based on existing WHO/ALERRT guidance and recommendations, while within one week Portugal had adopted new strategies to facilitate expedited assessment of COVID-19 research. India produced detailed national guidelines for its ethics committees by April 2020, and these were subsequently downloaded in over 45 countries At the operational level, some committees succeeded in achieving **very rapid turn-around times** for the review of COVID-related studies**.** Examples shared included: 2–3 days in Brazil; 2–5 days in Portugal; 3 days in Barbados; 5 days in Egypt, 7 days in South Africa, and 7 days in Iran. Approaches used either at strategic or operational level included:
• Adopting **more flexible systems,** including making effective use of email and online meetings, **and supporting more ad hoc communication** with Principal Investigators (PIs) to minimise time-wasting ‘back and forth’ between applicant and committee (many countries). Building on this, New Zealand is exploring pre-review of applications, sharing summarised initial comments from either the secretariat or the committee members with the PI in advance of meetings, with the aim of facilitating a more constructive dialogue in the meeting itself
• **Simplifying procedures and documentation** for multinational COVID-19 clinical trials with permission for incomplete submissions (for example lacking national documentation) to be updated at a later stage (Portugal)
**• Bringing different forms of expertise** into committees, ensuring that members had the necessary technological confidence to operate online, and allowing for alternate members in order to share the time commitment and enable rapid response (India)
• **Compensating committee members** to ensure availability of reviewers (Singapore)
• Improving **communication with regulatory bodies:** for example members of the Iranian National Research Ethics Committee participated in scientific review meetings, thus ensuring they had better understanding of the protocols before they came to the NREC, while for some studies a member of the national regulatory authority sat as an observer in NREC meetings
The opportunities offered by the Lisbon summit in **supporting regional as well as global cooperation** were also emphasised. Fifteen participants from the African Region, comprising representatives of the WHO regional office and members of some national ethics committees held a regional side-event to deliberate on support for the establishment of national ethics committees in all the countries in the region and the contributions African countries could give to support bioethics globally. It was resolved that a regional summit of national ethics committees should be held in the African Region for the first time, focusing on the theme: “Preparing African Research and Ethics for the next pandemic”

## Remaining challenges

Nevertheless, significant challenges remain: some arising directly out of the emergency context, and others illustrating how unresolved issues within ethical review (in particular how best to review multi-site and multi-country studies) arise in particularly acute form in emergencies. Common experiences from research ethics committees around the globe, shared at the Lisbon meeting, included:

*Extraordinary pressure on both national and local-level research ethics committees*, with very high numbers of applications to review, coupled with similarly high numbers of amendments to existing studies in order to comply with COVID-related public health restrictions. The intense nature of the working environment was compounded by competing professional commitments for those involved, social and researcher pressure to ‘do something’, and in some cases strong political pressures to approve studies regardless of ethical or scientific concerns. Such pressures lead to burn-out, particularly when sustained over an extended period.

*Inadequate resourcing*, over and above existing well-recognised limitations in many countries. The large increases in funding made available for research as part of the emergency response to COVID-19 did not trickle through to review processes, despite the enormous increase in the number of applications, and the expectations of rapid response.

*Capacity challenges*, particularly for committees with less experience of reviewing complex trial designs, innovative methodologies, and multi-country studies. Inevitably these challenges were particularly acute for committees that were already inadequately resourced and supported.

*Difficulties in negotiating the opportunity costs inherent in prioritising COVID-19 related studies*. While establishing a separate ad hoc committee for fast-track reviews could be helpful, identifying which studies should be expedited for review was not straightforward, especially given concerns as to how many registered clinical trials for COVID-19 interventions were inadequately powered to produce reliable results [13, 14]. The scope for potential research waste raises difficult questions for research ethics committees, and highlights the responsibilities of other stakeholders to act earlier in the research process to prioritise and co-ordinate research proposals from a public health perspective.

*The variable quality of studies*, including data and tissue management plans that lacked clarity or detail because of pressure of time. It was reported that at times inadequate expert scientific scrutiny before studies were submitted for ethical review increased the burden of scrutiny on research ethics committees.

*Co-ordinated review of multi-site studies in-country*: while some countries reported how such studies were referred directly to a designated committee (including, for example, in Brazil, Panama, Barbados, Egypt, New Zealand), national strategies of this kind were far from universally in place and implemented. As already recommended by PAHO and widely implemented in Latin America [15], there is an urgent need for all countries to devise and implement a national (or subregional, or provincial, as relevant) strategy to organize ethics review in emergencies, and allocate responsibilities, in ways that minimise duplication and delay, particularly for multi-site studies. Such strategies also need to be able to account for how ethical issues specific to particular sites will be appropriately handled.

*Co-ordinated review of multi-country studies*: where multi-site studies also involve multiple countries, ensuring effective and timely review remains even more challenging, including also scope for tension between national and WHO-level review. The African Vaccine Regulatory Forum (AVAREF) has developed a model, whereby delegates from a number of participating countries come together in a joint meeting to discuss a protocol, with decisions then being delivered by the relevant committee in each country within an agreed timescale, informed by a shared understanding of the proposal [16]. Alternative possible models include exploration of circumstances in which mutual recognition by ethics committees of each other’s judgments could be acceptable. More ambitiously still, future consideration could be given to the creation of regional committees (or even a global committee) with a specific mandate to review protocols for research in emergencies on behalf of participating countries, coupled with the option of local review with respect to site-specific factors. In order to be successful, such an initiative would, however, require willingness to achieve any necessary legislative change at the level of national jurisdictions. Research on different models of research ethics cooperation is urgently needed in order to foster innovation and evaluate different models. Such approaches also need to be sensitive to the heterogeneity and diverse contexts of different ethics committees.

## What is needed to improve preparedness for the next public health emergency?

Attendees identified four priorities to take forward a more ethically-prepared ethical review system (Table 2).

**Table 2:** Four priorities to take forward a more ethically-prepared ethical review system

1. Action by governments and national ethics bodies, supported by WHO, to **translate existing ethical guidance into practice**: through consolidating guidance from diverse sources; sharing good practice and tools effectively; and ensuring that systems and structures are in place to enable guidance to be implemented
2. **Critical (internal) scrutiny by the ethics community of what the ethical review system offers, and how it fulfils its remit**: through reviewing processes from the perspective of researchers; contributing to ethics education and awareness; making the case for the value that good review adds; exploring the development of quality indicators to support accountability; and using action research and evaluation to tackle longstanding issues in review processes
3. **Increased support for the capacity, skills and confidence** of research ethics committee members and staff, particularly with reference to novel trial designs: including through effective networking and the development of systems of mutual learning and support at both local and international level
4. **Adequate funding of ethical review infrastructure**: through ensuring that research ethics committees receive a set percentage of research funding upfront so that they operate their functions effectively and contribute to the shared aim of facilitating ethically-conducted research

First, all agreed that ‘more guidance’ was not required at this point, other than in connection with specific issues such as novel study designs [17]. Rather, action is required by multiple stakeholders to translate existing guidance into practice. The recommendations produced in 2018 as a result of the meeting co-hosted by WHO and ALERRT [1], for example, proved to be highly relevant in responding to the challenges of COVID-19 alongside further regional-level guidance reiterating the importance of national strategies [8]. The rapid and flexible turnaround achieved by some committees confounds the myth that ethical review is necessarily a stumbling block to rapid research.

Nevertheless, much remains to be done on a global scale to consolidate existing guidance from diverse sources (as PAHO has already done for its stakeholders in the Americas [10]); to share the good practice and tools developed by many ethics committees; and to achieve the systems and structures necessary to act on those recommendations, with diverse stakeholders bearing responsibility for different aspects (see Table 3 below). In particular, all these stakeholders have a role to play in ensuring that countries have in place models of multi-country review that provide effective, responsive review, and meet each country’s needs for accountability and control, while minimising duplication of effort and delay.

**Table 3:** Stakeholders with responsibilities to contribute to emergency preparedness in ethical review

**• National governments/ departments of health** are responsible for ensuring there is a clear strategy for ethical review within their jurisdiction, setting out its relationship with other elements of research governance and oversight systems, such as scientific review and regulatory bodies. Such a strategy is an essential pre-requisite for the development of streamlined systems that facilitate effective co-ordination between these different elements. As attendees illustrated, different strategic models will be suitable for different countries and contexts, depending on the size of the research sector, the nature of the studies being reviewed, and the level of expertise available. Governments are also responsible for ensuring that human and financial resources are rapidly made available to meet the increased demands on ethics committees at the height of an emergency
• Where countries have **national research ethics bodies** with a remit from national government to oversee the operation of ethical review, their designated responsibilities may include developing emergency SOPs that will facilitate rapid and responsive ethical review in emergencies, contributing, where appropriate, to regional initiatives to achieve co-ordinated review of multi-country studies, and making best use of available capacity
• The responsibilities of **WHO (Regional Offices and the headquarters Health Ethics and Governance Unit)** include working with national research ethics bodies and other relevant stakeholders to help support clearer lines of communication, networking and mutual learning; promote the rapid dissemination of good practice guidance; and help minimise duplication of efforts. One proposal put forward at the meeting was the establishment of an online platform to connect national research ethics committees and provide a focal point for relevant guidance. Collaboration between and within WHO departments, and with those concerned with regulatory systems, is also crucial

Second, critical (internal) scrutiny is required of what the ethical review system offers, and how it fulfils its remit. Such scrutiny and action needs to take place during inter-pandemic periods, in order to support better emergency preparedness for the future. It was argued that there is an onus on everyone involved in ethical review to make the case for the value that the review process can bring in prompting explicit consideration of the values that should inform decision-making during the entire research process. Research ethics committees will be much better placed to achieve this outcome if they are perceived not as a negative, adversarial, hurdle in the research system, but rather as a contributor in the production of better, socially-valuable, research. This recognition that everyone concerned in research shares responsibilities for the ethical conduct of that research is particularly important in the testing circumstances of a public health emergency. Alongside building on the more flexible and responsive approaches developed during the pandemic, such a shift in perception will require:

- Genuine willingness on the part of ethics committees to test themselves as to how they add value – including reviewing their own processes from the point of view of researchers, sponsors and others.
- Recognizing the need for those involved in bioethics to take teaching research ethics seriously, so that researchers of the future have a better understanding of the ethical aspects of their work, and what value ethical review can bring. In addition to contributing to undergraduate teaching within health sciences curricula (medicine, pharmacy, dentistry, nursing, allied health sciences, etc.), this could involve taking part in initiatives such as roadshows to improve knowledge and understanding of ethical review, providing training materials, and using newsletters and social media platforms to raise the profile of ethical review.
- Making the case proactively to researchers for how timely identification of ethical concerns actively benefits research outcomes – for example how making the case for equitable and inclusive recruitment practices may lead to tangible benefits such as greater confidence and trust in novel interventions when they are translated into practice [18].
- Exploring the development of quality indicators to help cement trust between and within committees, researchers and other research stakeholders, and help identify, at the level of individual committees, where changes in practice would be beneficial. Such indicators could build on current work by WHO on benchmarking tools for ethics committees [19], and on a regional initiative by PAHO on indicators for core components of research ethics systems [20], to capture both substantive elements (the value added to the study as a result of ethical scrutiny) and valued performance elements such as flexibility and timeliness. Examples of initiatives in this area shared by meeting attendees included the development of performance indicators in Egypt; while in New Zealand, a new ‘decision analyses’ process will be used to identify what ethical issues most frequently lead to applications requiring revision, and then consider whether more training or guidance is required, or whether the standards themselves require review.
- Seeking funding for research into research ethics: using applied research and quality improvement processes to identify and solve persistent problems in research ethics processes. Key questions for such research to address would include: substantive elements such as understanding of optimal consent and community engagement processes, particularly in connection with novel research approaches; procedural questions, such as optimal training and composition of committees; and perceptual/relational issues regarding how relationships between committees and researchers could be improved [21].

Third: action to support capacity, skills and confidence, particularly with reference to complex and innovative trial designs. The need to support mutual learning, at both local and international level, was strongly emphasised by attendees, with suggestions including the creation of a ‘learning research ethics committee system’ (echoing the concept of a learning healthcare system) and the development of a ‘community of practice’ for mutual learning among research ethics committee members. Such approaches highlight again the important role of effective networking between ethics committee members, and the scope for WHO at both regional and global level to support this.

Fourth: None of this can be achieved without adequate funding of ethical review infrastructure. Ethics committees need a permanent office, space, staff, infrastructure (including increasingly sophisticated online platforms and software) and budget to run their functions and contribute effectively to the shared aim of facilitating ethically-conducted research. Consideration must also be given to when and how it might be appropriate to compensate committee members, recognising that relying on unpaid labour to carry out a critical part of the research oversight system is not only unsustainable at these levels of pressure but also carries a message about how that labour, and the skills required to conduct it, are valued [12]. Recognising all these factors, attendees felt that it is time for research ethics infrastructure to receive a set percentage of research funding, levied at source, and allocated in such a way as to provide for a sustainable committee system that has the time, capacity and skills to contribute consistently to the improvement of research proposals.

## Conclusion

Investment in ethical preparedness in the years preceding the emergence of the COVID-19 pandemic enabled research ethics committees in many parts of the world to contribute flexibly and promptly to the global research-led emergency response. Significant challenges, however, remain, in light of the extreme pressure placed on ethical review systems by factors including high numbers of emergency research proposals, testing timeframes, political exigencies, inadequate infrastructure and support, and the inherent complexities of multi-site, multi-country studies.

In order to be able to respond effectively in future emergencies, research ethics committees need to be better prepared, better resourced, and better connected, both with each other and with the rest of the research ecosystem. They need to be recognized as offering added value for researchers and funders, helping design better studies and improving respect for participants’ rights, instead of being perceived as an obstacle to innovation and research development. When research ethics committees are seen as an essential part of the whole research process then, as a society, we will be better prepared to face the next emergency.

Diverse stakeholders, including national governments, national and local research ethics bodies, WHO, research funders, and researchers themselves, all have their part to play in achieving such a transformation. In particular, as an ethics community, we need to be better at consolidating guidance and translating it into practice; we need to enhance our relationships with other parts of the research ecosystem, including the transparency with which we operate; we need to be willing to reform and adapt our systems and processes; and we need to make the case robustly that better resourcing is key.

## Data Availability

Not applicable.
